# Taxonomic study of the leafhopper genus *Oncopsis* (Hemiptera, Cicadellidae, Macropsinae) from Sichuan Province, China with description of two new species and a key to males

**DOI:** 10.3897/zookeys.854.33117

**Published:** 2019-06-10

**Authors:** Hu Li, Juan Li, Ren-Huai Dai

**Affiliations:** 1 Shaanxi Key Laboratory of Bio-resources, School of Biological Science & Engineering, Shaanxi University of Technology, Hanzhong, Shaanxi, 723000 China Institute of Entomology of Guizhou University Guizhou China; 2 Institute of Entomology of Guizhou University, The Provincial Key Laboratory for Agricultural Pest Management of Mountainous Region, Guiyang, Guizhou, 550025 China Shaanxi University of Technology Shaanxi China

**Keywords:** Auchenorrhyncha, China, distribution, morphology, taxonomy

## Abstract

This paper deals with the leafhopper genus *Oncopsis* (Macropsinae) from Sichuan Province of China, and describes and illustrates two new species, *O.konkaensis***sp. nov.** from Minya Konka (Sichuan), and *O.moxiensis***sp. nov.** from Moxi Town (Sichuan), and provides a key to males and a geographic distribution map for *Oncopsis* species from Sichuan.

## Introduction

The leafhopper genus *Oncopsis* Burmeister, 1838 includes more than 90 members (Dai et al. 2018, Li et al. 2018) around the world, and is the second largest group in the subfamily Macropsinae (Hemiptera: Cicadellidae). *Oncopsis* has been treated as a tribe of the subfamily Eurymelinae recently (Dietrich and Thomas 2018), and has a distribution mostly in the Holarctic region. The type species is *Cicadaflavicollis* Linnaeus, 1761. *Oncopsis* differs from other macropsine genera in having the face with coronal pits closer together than the ocelli, the usually transversely striate pronotum, the male pygofer without a process, and the s-shaped male dorsal connective that is usually produced into various processes from its inner ventral margin.

Almost all species of *Oncopsis* are oligophagous or monophagous on Betulaceae, including *Betulaprocurva* Litv., *B.turkestanica* Litv., *Alnusbarbata* C.A.Mey., *A.hirsuta* (Spach) Rupr., *A.japonica* (Thunb.) Steud., *Duschekia* spp., and *Carpinusbetulus* L. (Tishechkin 2016). Only one species, *Oncopsiskrios* Mühlethaler, is an exception and is associated with *Ulmus* sp. (Ulmaceae) (Mühlethaler 2008). Sichuan Province is located in the Qinghai-Tibet, southwest and central China regions under the divisions of Zoogeographical Regions of China (Chen 1997), a key area for insect biodiversity. The first species of *Oncopsis* recorded in China, *O.fusca* (Melichar, 1902), was reported from Sichuan Province. Later, Xu et al. (2006), Dai and Li (2013), Kuoh (1992), Li et al. (2018) and Dai et al. (2018) described new species or reported *Oncopsis* from this area. To date, 14 species of *Oncopsis*, including the two new species described here, are known from Sichuan Province, which has more than 40% of the total number (n = 33) of *Oncopsis* species distributed in China (Dai et al. 2018, Li et al. 2018).

In the present paper, the genus *Oncopsis* from Sichuan Province, China is reviewed, and two new species, *O.konkaensis*, sp. nov. from Minya Konka and *O.moxiensis*, sp. nov. from Moxi Town, are described and illustrated. A geographic distribution map and a key for identification of *Oncopsis* from Sichuan Province (based on male features) are provided.

## Materials and methods

Specimens were collected by sweep net. External morphology was observed under an Olympus SZX7 and BX43 microscopes. Male genitalia preparations were made by placing the whole abdomen in a boiling solution of 8% NaOH for 5 minutes, then rinsing with fresh water several times and transferring into glycerin on glass slides for examination, dissection, drawing, and photography. The dissected genitalia and remains of the abdomen were stored in micro vials containing glycerin for further examination.

Habitus images of adults were obtained with an Olympus SZX7 microscope associating with a Canon EOS 550D camera. Genitalia drawings were made and edited with Adobe Illustrator CS6 and Photoshop CS6.

The morphological terminology used in this work for the species descriptions follow the works of Anufriev (1967), Hamilton (1980), and Tishechkin (2017). The body length was measured from the apex of the head to the end of the forewings and is given in millimeters.

The type specimens of the new species are deposited in the Museum of Zoology and Botany, Shaanxi University of Technology, Hanzhong, China (**SUHC**), and the other examined specimens are deposited in the Institute of Entomology, Guizhou University, Guiyang, China (**GUGC**).

## Taxonomy

### Genus Oncopsis

Taxon classificationAnimaliaHemipteraCicadellidae

Burmeister, 1838

Bythoscopus (Oncopsis) Burmeister, 1838: 10.
Zinneca Amyot & Servile, 1843: 579; Hamilton 1980: 887 (synonymy).

#### Type species.

*Cicadaflavicollis* Linnaeus, 1761 [by subsequent designation, Westwood 1840].

#### Distribution.

Palaearctic, Oriental, and Nearctic realms.

#### Host.

Betulaceae and *Ulmus* spp. (Ulmaceae).

#### Remarks.

*Oncopsis* can be distinguished from other genera of Macropsinae largely by the following combined features: face with coronal pits closer together than ocelli; frons usually with transverse striations or punctures; pronotum with transverse striations; forewing with three (rarely two or reticulate) anteapical and four apical cells; male pygofer without process at ventral margin; dorsal connective generally large, s-shaped in lateral aspect, and bearing large, forked or unforked process from inner ventral margin; dorsal connective usually articulating against upper margin of pygofer.

### Oncopsis anchorous

Taxon classificationAnimaliaHemipteraCicadellidae

Xu, Liang & Li, 2006

Oncopsis anchorous Xu, Liang & Li, 2006: 836

#### Material examined.

1 male [Holotype], 1 male and 1 female [Paratypes]: CHINA: Sichuan Province, Emeishan, 16-vii-1995, collected by Mao-Fa Yang (GUGC).

#### Distribution.

Sichuan (Fig. 65).

### Oncopsis furca

Taxon classificationAnimaliaHemipteraCicadellidae

Liu & Zhang, 2003

Oncopsis furca Liu & Zhang, 2003: 181

#### Material examined.

1 male: CHINA: Sichuan Province, Tibetan Autonomous Prefecture of Garzê, Luding County, Moxi Town, Hailuogou, 3000 m above sea level, 29-vii-2012, collected by Meng Jiao (GUGC).

#### Distribution.

Sichuan (Fig. 65), Gansu, and Qinghai (Dai et al. 2018, Li et al. 2018).

### Oncopsis fusca

Taxon classificationAnimaliaHemipteraCicadellidae

(Melichar, 1902)

Bythoscopus fuscus Melichar, 1902: 120
Oncopsis fusca Metcalf 1966: 219; Lauterer and Anufriev 1969: 162

#### Material examined.

None.

#### Distribution.

Sichuan (Fig. 65), Tibet, and Hubei; Philippines, and Malaysia (Dai et al. 2018, Li et al. 2018).

### Oncopsis graciaedeagus

Taxon classificationAnimaliaHemipteraCicadellidae

Li, Dai & Li, 2018

Oncopsis graciaedeagus Li, Dai & Li, 2018: 31

#### Material examined.

1 male [Holotype], 5 males and 3 females [Paratypes]: CHINA: Sichuan Province, Tibetan Autonomous Prefecture of Garzê, Luding County, Moxi Town, Hailuogou, 3000 m above sea level, 29-vii-2012, collected by Hu Li, Zhi-Hua Fan, and Meng Jiao (GUGC).

#### Distribution.

Sichuan (Fig. 65).

### Oncopsis hailuogouensis

Taxon classificationAnimaliaHemipteraCicadellidae

Li, Dai & Li, 2018

Oncopsis hailuogouensis Li, Dai & Li, 2018: 33

#### Material examined.

1 male [Holotype]: CHINA: Sichuan Province, Tibetan Autonomous Prefecture of Garzê, Luding County, Moxi Town, Hailuogou, 3000 m above sea level, 29-vii-2012, collected by Meng Jiao (GUGC).

#### Distribution.

Sichuan (Fig. 65).

### Oncopsis kangdingensis

Taxon classificationAnimaliaHemipteraCicadellidae

Dai & Li, 2013

Oncopsis kangdingensis Dai & Li, 2013: 12

#### Material examined.

1 male [Holotype], 1 male and 7 females [Paratypes]: CHINA: Sichuan Province, Tibetan Autonomous Prefecture of Garzê, Kangding County, 2700 m above sea level, 10-viii-2010, collected by Yi Tang (GUGC).

#### Distribution.

Sichuan (Fig. 65), Shanxi, and Yunnan (Dai et al. 2018, Li et al. 2018).

### Oncopsis konkaensis

Taxon classificationAnimaliaHemipteraCicadellidae

Li, Li & Dai sp. nov.

http://zoobank.org/4763F5C2-7588-4B82-A7F2-78256B2E2162

Figs 1–3 , 7–16 , 65

#### Type material.

***Holotype male*** : CHINA: Sichuan Province, Tibetan Autonomous Prefecture of Garzê, Luding County, Minya Konka, Yajiageng, 3800 m above sea level, 13-viii-2015, collected by Hong-Ping Zhan (GUGC).

#### Etymology.

The specific epithet was derived from the type locality, Minya Konka (Sichuan Province), where the species was collected, combined with the Latin suffix -ensis, meaning from a locality.

#### Description.

**Figures 1–6.**
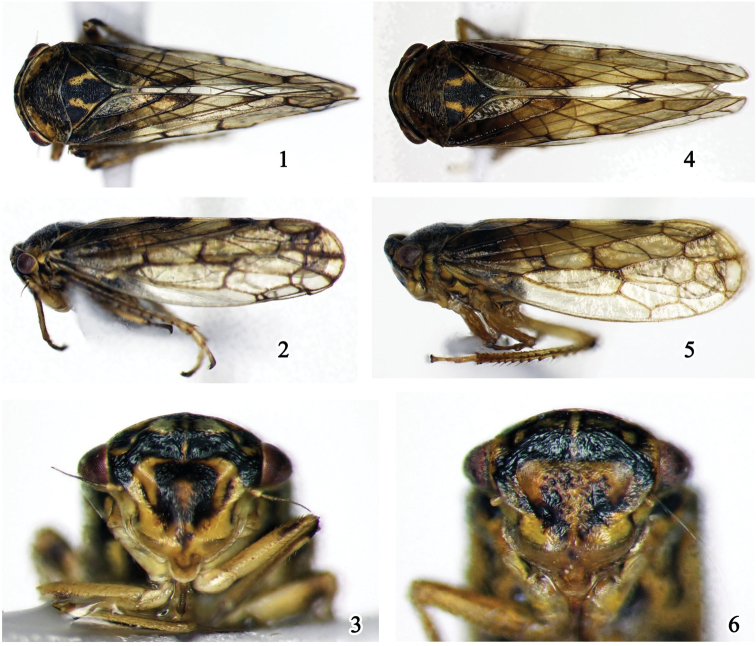
Males of *Oncopsis* in dorsal (**1, 4**), and lateral (**2, 5**) views, and face (**3, 6**) **1–3***O.konkaensis* sp. nov. **4–6***Oncopsismoxiensis* sp. nov.

 [Holotype] ***Body color.*** Body background color (Figs 1, 2) yellowish. Crown (Fig. 1) with black transverse stripe. Face (Fig. 3) yellow, eyes reddish brown; antenna with pedicel and scape yellowish brown and flagellum dark brown; frons with approximately m-shaped black macula between eyes; frontoclypeus with n-shaped black macula at middle with two ends close to each other, and dark oblique striation near lateral margin; clypeus with brown markings. Pronotum (Fig. 1) dark brown medially, lighter anterolaterally. Scutellum (Fig. 1) black with pair of posteriorly diverging yellow submedial stripes. Forewing (Figs 2, 3) pale hyaline infused with brown, venation dark brown. Legs yellowish, marked with brown maculae.

***Body appearance.*** Typically wedge-shaped. Head (Fig. 1) short, with parallel margins, broadly convex in dorsal view; width across eyes as wide as pronotum. Face including eyes (Fig. 3) slightly wider than long, distance between ocelli nearly 4 × that from ocellus to adjacent eye, frons with distinct rugae and longitudinal carina, clypeus with few scattered punctures. Pronotum (Fig. 1) with obvious closely-spaced transverse striations, anterior margin prominent frontally, and posterior margin concave medially, broader by 2.6 × length. Scutellum (Fig. 1) triangular, with coarse surface, middle length 1.5 × that of pronotum. Forewing (Figs 2, 3) hyaline, with three anteapical and four apical cells, veins well defined.

Male abdominal apodemes of second tergite (Fig. 9) weakly sclerotized, with rounded apex. Apodemes of second sternite (Fig. 10) basally broad, tapered to subacute apex, and pointed towards each other, distance between apodemes nearly 2 × their middle length.

**Figures 7–16.**
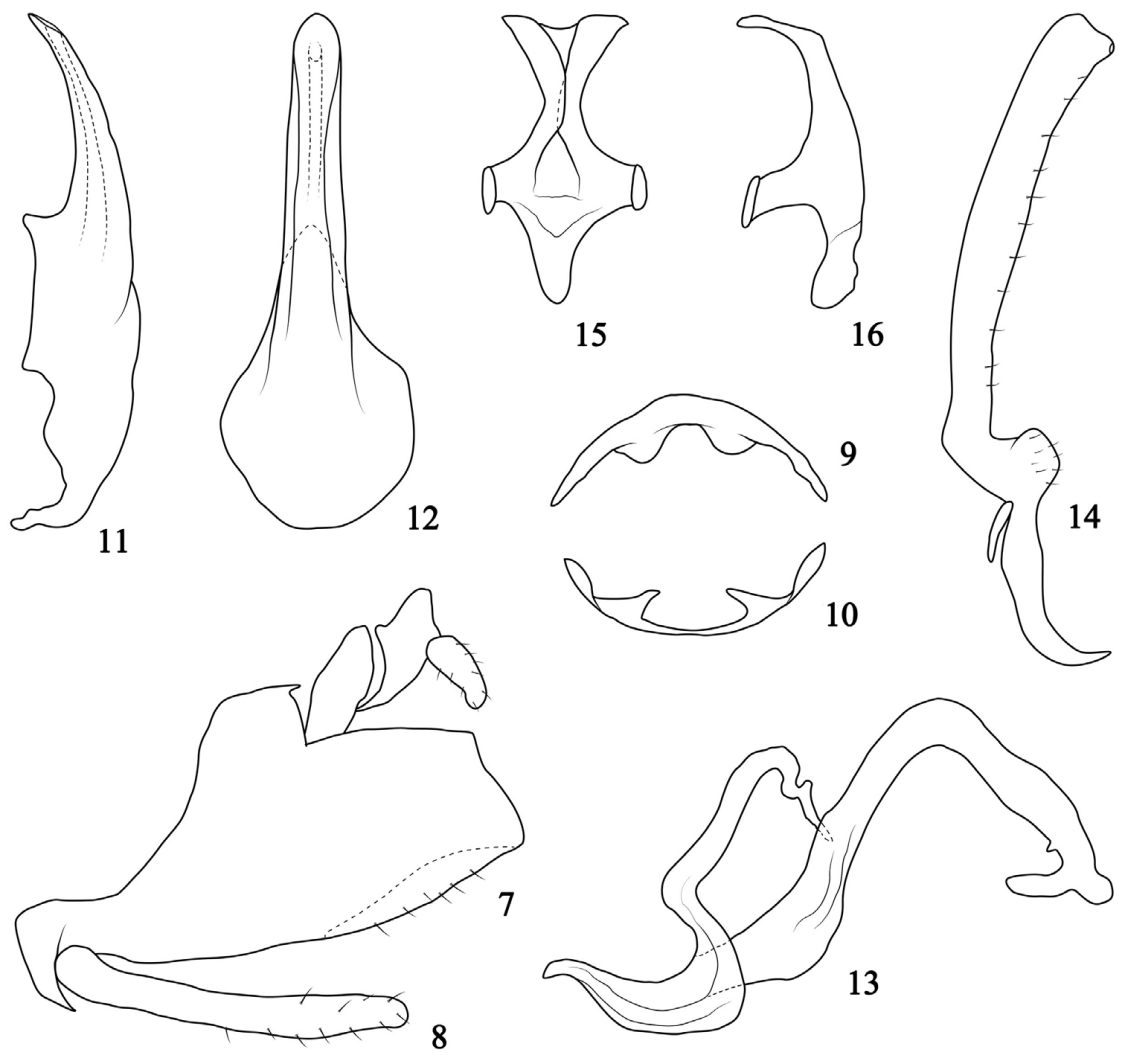
*Oncopsiskonkaensis* sp. nov. **7** Male pygofer, lateral view **8** Subgenital plate, lateral view **9** 2^nd^ abdominal tergal apodemes **10** 2^nd^ abdominal sternal apodemes **11** Aedeagus, later view **12** Aedeagus, ventral view **13** Dorsal connective, lateral view **14** Style, dorsal view **15** Connective, dorsal view **16** Connective, lateral view.

***Male genitalia.*** Pygofer side broad basally (Fig. 7), dorsal and caudal margin truncated, ventral margin with distal half expanded inwards, with scattered setae. Subgenital plate (Fig. 8) slender, 0.6 × length of ventral margin of pygofer. Aedeagus (Figs 11, 12) with broad basis, slender shaft, tapered to subacute end in lateral aspect, margins somewhat parallel, with round apex in ventral view, gonopore subapical. Dorsal connective (Fig. 13) s-shaped in lateral view, produced to large and long process from inner ventral margin bent ventrad beyond mid-length, apex bifurcate. Style (Fig. 14) with stout stem, dorsally bent, gradually widening to apex, with marginal setae, apical margin truncated. Connective (Figs 15, 16) typical of the genus.

#### Measurement.

Body length (including tegmen): 5.0 mm.

#### Distribution.

Sichuan (Fig. 65).

#### Host.

*Betula* spp. (Betulaceae).

#### Remark.

The new species differs from all other known members of *Oncopsis* by the unique shape of the dorsal connective, which has the medial process large and long, bent ventrad and bifurcated at the apex; also by the combined features of the aedeagus and pygofer.

### Oncopsis kuluensis

Taxon classificationAnimaliaHemipteraCicadellidae

Viraktamath, 1996

Oncopsis kuluensis Viraktamath, 1996: 185; Dai and Li 2013: 17.

#### Material examined.

3 males: CHINA: Sichuan Province, Emeishan National Natural Reserve, Jinding, 7-viii-1991, collected by Zi-Zhong Li (GUGC); 2 females: CHINA: Sichuan Province, Emeishan National Natural Reserve, Leidongping, 7-viii-1991, collected by Zi-Zhong Li (GUGC).

#### Distribution.

Sichuan (Fig. 65) and India (Viraktamath 1996, Li et al. 2018).

### Oncopsis ludingensis

Taxon classificationAnimaliaHemipteraCicadellidae

Li, Dai & Li, 2018

Oncopsis ludingensis Li, Dai & Li, 2018: 36.

#### Material examined.

1 male [Holotype], 1 male and 5 females [Paratypes]: CHINA: Sichuan Province, Tibetan Autonomous Prefecture of Garzê, Luding County, Moxi town, Hailuogou, 3000 m above sea level, 29-vii-2012, collected by Li Hu, Fan Zhi-Hua and Jiao Meng (GUGC).

#### Distribution.

Sichuan (Fig. 65).

### Oncopsis melichari

Taxon classificationAnimaliaHemipteraCicadellidae

Lauterer & Anufriev, 1969

Oncopsis melichari Lauterer & Anufriev, 1969: 163.

#### Material examined.

None.

#### Distribution.

Sichuan. Note: the distribution of *O.melichari* is excluded from the distribution map since the collected data, “the valley of the river Shubagu” of the original record (Lauterer and Anufriev 1969), cannot be matched with any known place names.

### Oncopsis moxiensis

Taxon classificationAnimaliaHemipteraCicadellidae

Li, Li & Dai sp. nov.

http://zoobank.org/224A1FE9-23CE-465F-8D22-A3BF6803BFDA

Figs 4–6 , 17–26 , 65

#### Type material.

***Holotype male*** : CHINA: Sichuan Province, Tibetan Autonomous Prefecture of Garzê, Luding County, Moxi Town, Hailuogou, 3600 m above sea level, 12-viii-2015, collected by Hong-Ping Zhan (GUGC).

#### Etymology.

The specific epithet was derived from place name, Moxi Town, where the species was collected and the type locality is located, combined with the Latin suffix -ensis, meaning from a locality.

#### Description.

[Holotype] ***Body color.*** Background yellow brown. Crown (Fig. 4) dark brown. Face (Fig. 6) yellow brown to dark brown, eyes brown, marked with reddish; antenna yellowish brown; frons dark to black except on ocelli and middle line; clypeus with central area dark or black on both sides of middle line, distal half chocolate. Pronotum (Fig. 4) dark brown with evenly dispersed darker spots. Scutellum and legs coloration similar to *O.konkaensis* sp. nov. Forewing (Figs 5, 6) with basal half dark brown and distal half yellowish brown.

***Body appearance.*** Relatively stout. Head including eyes (Fig. 4) slightly narrower than pronotum. Face across eyes (Fig. 6) broader than long, central region with obvious punctures. Pronotum (Fig. 4) 2.5 × wider than long, with fore-margin strongly protruding forward, and hind margin slightly depressed in middle. Scutellum (Fig. 4) 1.2 × longer than pronotum. Other features as in *O.konkaensis* sp. nov.

Male abdominal apodemes of second tergite (Fig. 19) broad, close to each other, twisted caudally. Apodemes of second sternite (Fig. 20) relatively small, basally broad, tapered to acute or subacute apex, and pointed inwards; distance between apodemes nearly 3 × their middle length.

**Figures 17–26.**
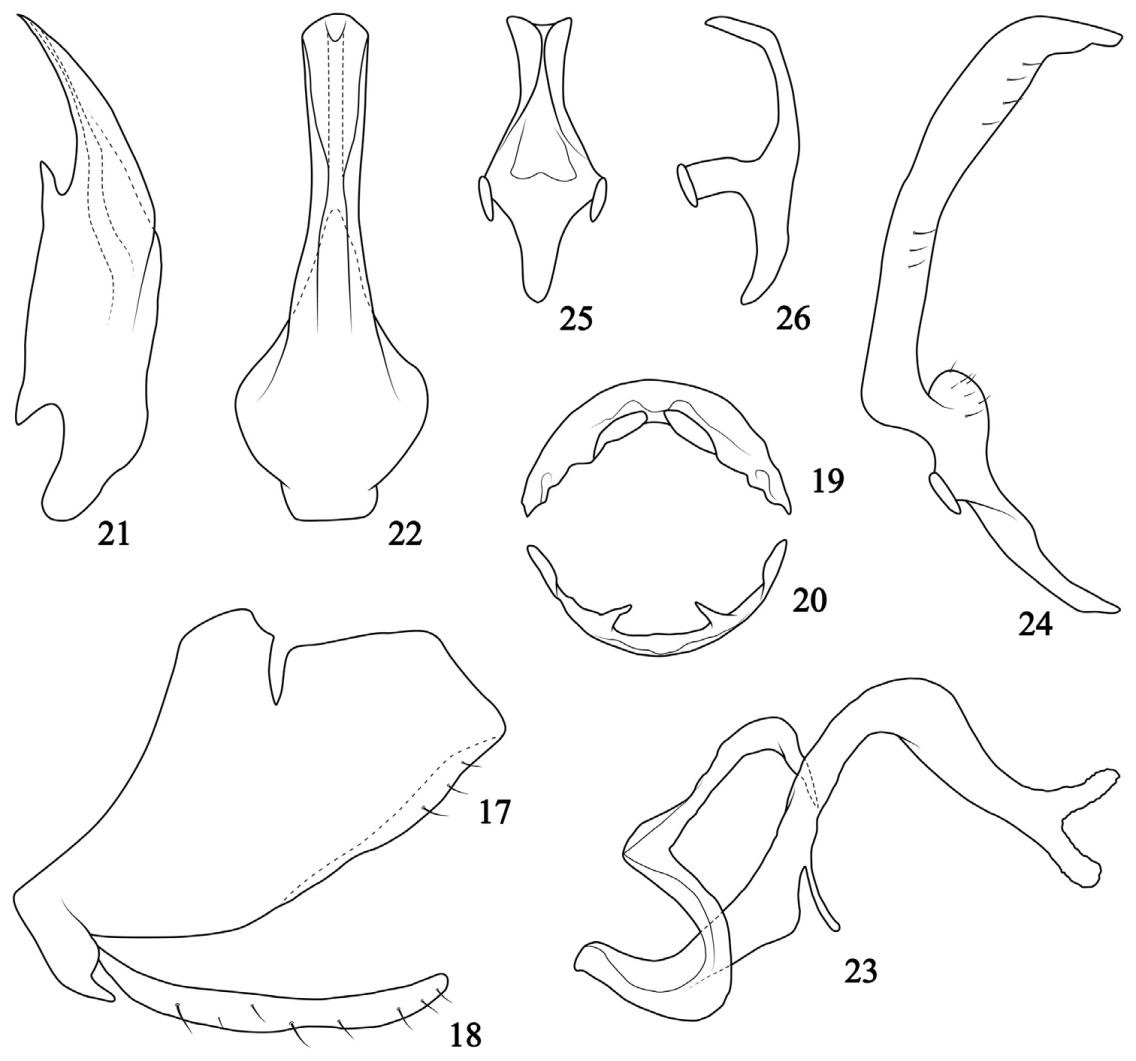
*Oncopsismoxiensis* sp. nov. **17** Male pygofer, lateral view **18** Subgenital plate, lateral view **19** 2^nd^ abdominal tergal apodemes **20** 2^nd^ abdominal sternal apodemes **21** Aedeagus, later view **22** Aedeagus, ventral view **23** Dorsal connective, lateral view **24** Style, dorsal view **25** Connective, dorsal view **26** Connective, lateral view.

**Figures 27–47.**
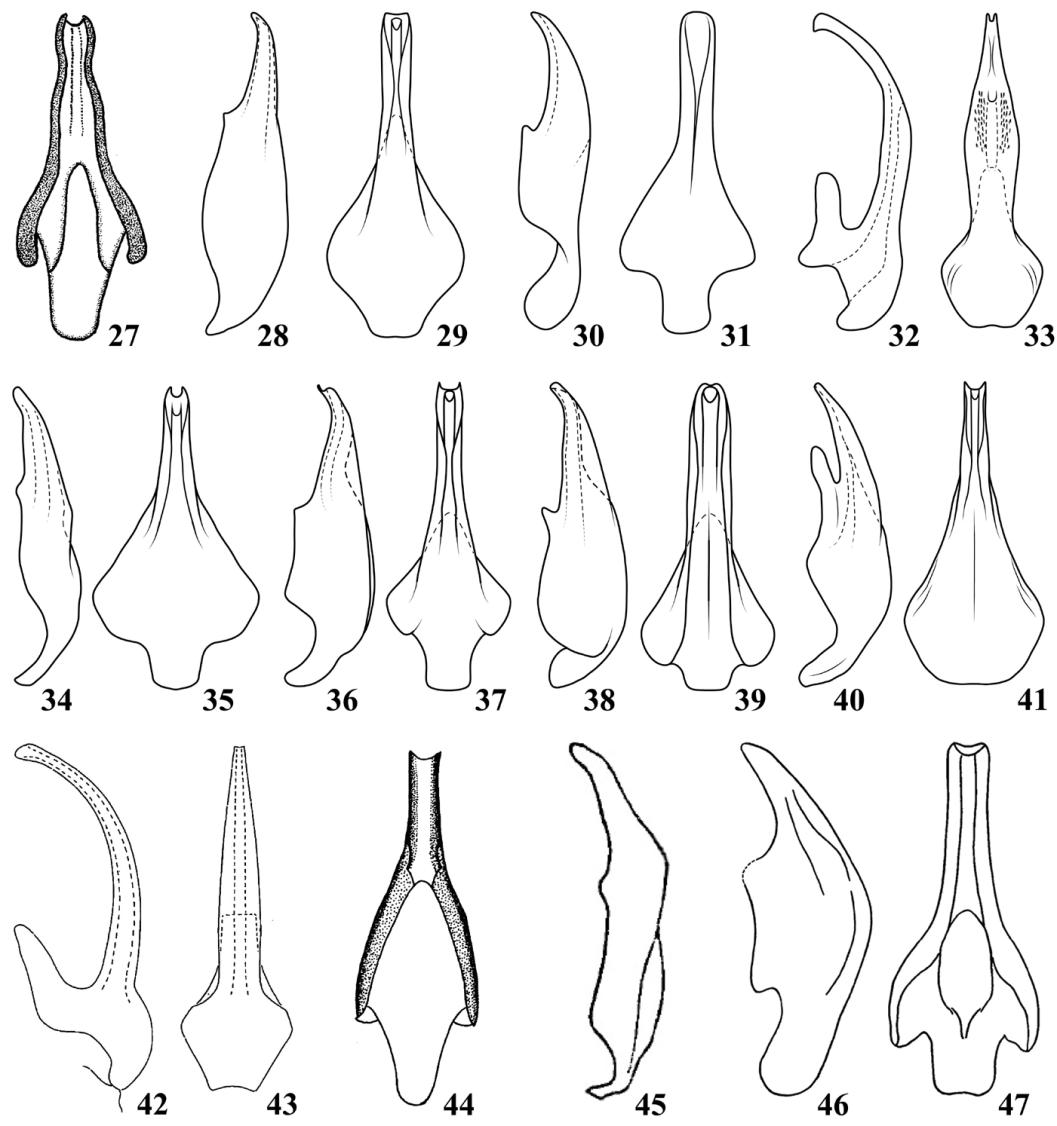
Aedeagus of *Oncopsis* in Sichuan, lateral (**28, 30, 32, 34, 36, 38, 40, 42, 45–46**) and ventral (**27, 29, 31, 33, 35, 37, 39, 41, 43–44, 47**) views **27***O.anchorous* (after Xu et al. 2006) **28–29***O.furca***30–31***O.fusca* (after Lauterer and Anufriev 1969) **32–33***O.graciaedeagus***34–35***O.hailuogouensis***36–37***O.kangdingensis***38–39***O.kuluensis***40–41***O.ludingensis***42–43***O.melichari* (after Lauterer and Anufriev 1969) **44***O.nigrofasciata* (after Xu et al. 2006) **45***O.trimaculata* (after Kuoh 1992) **46–47***O.tristis* (after Tishechkin 2017).

**Figures 48–64.**
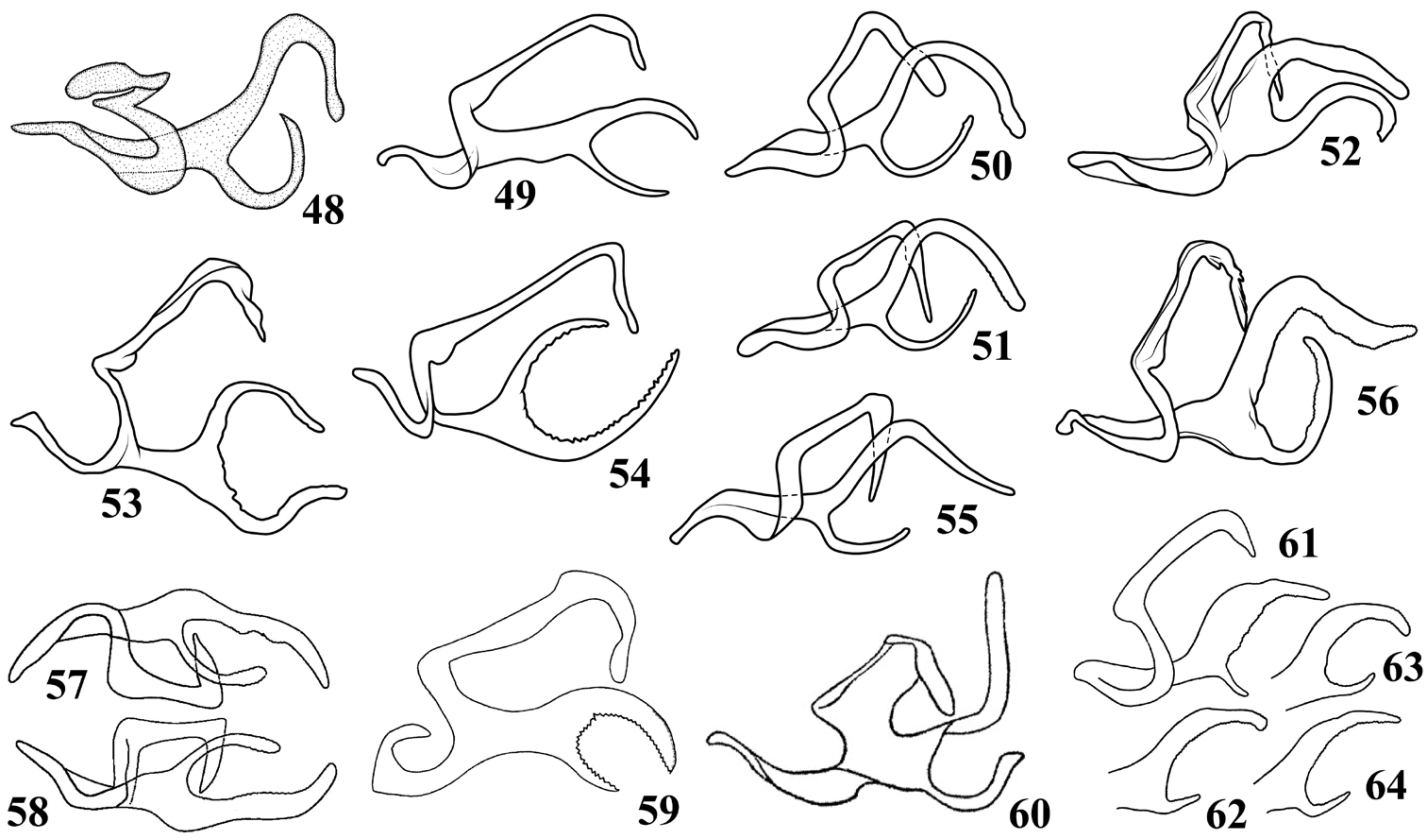
Dorsal connectives of *Oncopsis* in Sichuan, lateral views **48***O.anchorous* (after Xu et al. 2006) **49***O.furca***50–51***O.fusca* (after Lauterer and Anufriev 1969) **52***O.graciaedeagus***53***O.hailuogouensis***54***O.kangdingensis***55***O.kuluensis***56***O.ludingensis***57–58***O.melichari* (after Lauterer and Anufriev 1969) **59***O.nigrofasciata* (after Xu et al. 2006) **60***O.trimaculata* (after Kuoh 1992) **61–64***O.tristis* (after Tishechkin 2017).

***Male genitalia.*** Pygofer side (Fig. 17) basally broad, dorsal and caudal margins straight. Subgenital plate (Fig. 18) approximately 2/3 length of pygofer ventral margin. Aedeagus (Figs 21, 22) broad basally, shaft tapered to acute apex in lateral view, slightly narrowed in middle, apex rounded in ventral aspect, gonopore apical. Dorsal connective (Fig. 23) with large process bent ventrocaudally from inner ventral margin with bifurcated end and sinuated margins; with extremely slender process pointed ventrad near base. Style apex bent dorsad and irregularly tapered (Fig. 24); connective (Figs 25, 26) typical.

#### Measurement.

Body length (including tegmen): 5.4 mm.

#### Distribution.

Sichuan (Fig. 65).

**Figure 65.**
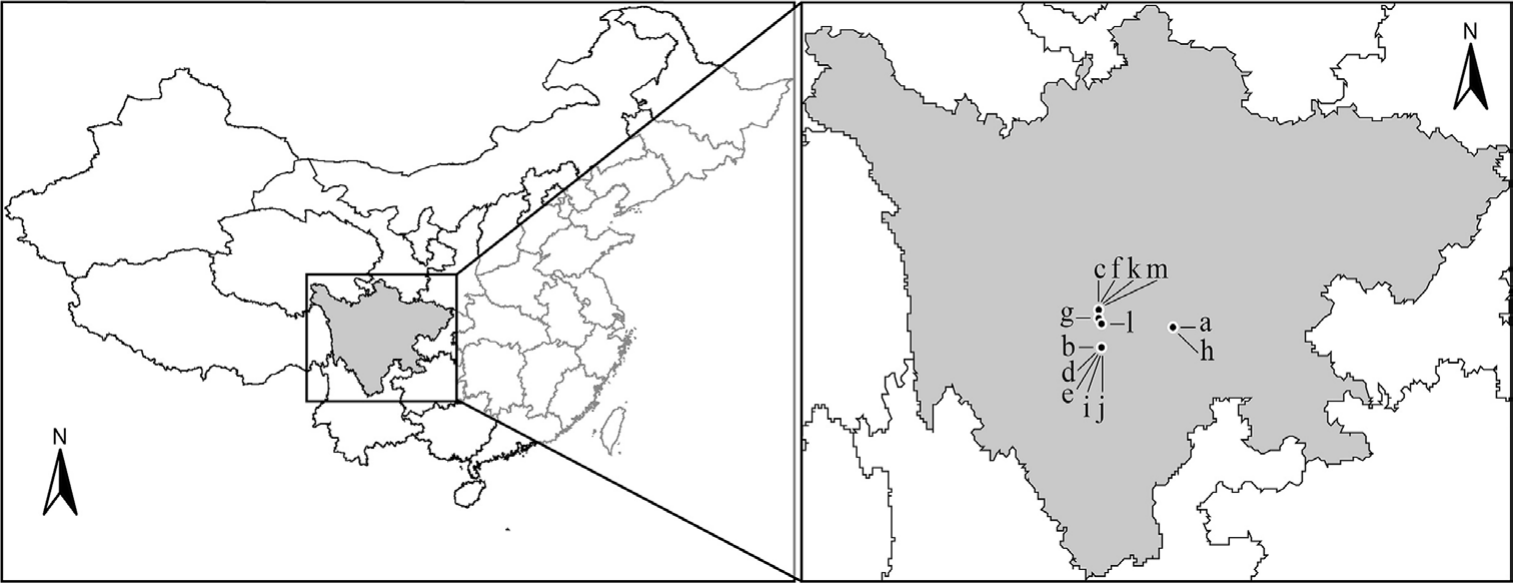
Map showing the distribution of species of *Oncopsis* in Sichuan Province, China. Key: a. *O.anchorous*; b. *O.furca*; c. *O.fusca*; d. *O.graciaedeagus*; e. *O.hailuogouensis*; f. *O.kangdingensis*; g. *O.konkaensis*; h. *O.kuluensis*; i. *O.ludingensis*; j. *O.moxiensis*; k. *O.nigrofasciata*; l. *O.trimaculata*; m. *O.tristis*.

#### Host.

*Betula* spp. (Betulaceae).

#### Remark.

This species is similar to *Oncopsiskonkaensis* sp. nov. in the body coloration and external morphology, and somewhat similar in the shape of the dorsal connective, but can be distinguished from the latter by the different coloration of the face, and the shapes of the aedeagus, style and the dorsal connective.

**Figures 66–68.**
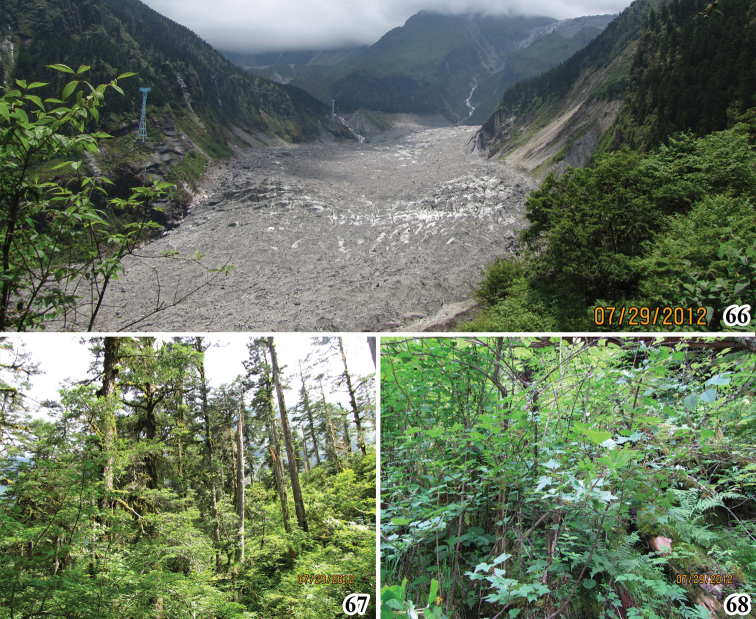
Photographs showing the landscape and *Oncopsis* habitat at Hailuogou of Sichuan **66** a tip of the Hailuogou glacier **67** Vegetation **68** Potential host plant to *Oncopsis*.

### Oncopsis nigrofasciata

Taxon classificationAnimaliaHemipteraCicadellidae

Xu, Liang & Li, 2006

Oncopsis nigrofasciatus Xu, Liang & Li, 2006: 837.
Oncopsis nigrofasciata , Dai, Li and Li 2018: 130 (correction of gender of species name).

#### Material examined.

1 male: CHINA: Sichuan Province, Tibetan Autonomous Prefecture of Garzê, Kangding County, 2700 m above sea level, 10-viii-2005, collected by Yi Tang (GUGC); 1 female: CHINA: Sichuan Province, Tibetan Autonomous Prefecture of Garzê, Kangding County, 23-vii-2012, collected by Zhi-Hua Fan (GUGC).

#### Distribution.

Sichuan (Fig. 65), Qinghai, Ningxia, Shanxi, Hebei, Yunnan, Shaanxi, and Jilin (Dai et al. 2018; Li et al. 2018).

### Oncopsis trimaculata

Taxon classificationAnimaliaHemipteraCicadellidae

Kuoh, 1992

Oncopsis trimaculata Kuoh, 1992: 272.

#### Material examined.

None.

#### Distribution.

Sichuan (Fig. 65).

### Oncopsis tristis

Taxon classificationAnimaliaHemipteraCicadellidae

(Zetterstedt, 1840)

Jassus tristis Zetterstedt, 1840: 303.
Oncopsis tristis , Metcalf 1966: 231; Lauterer and Anufriev 1969: 165; Tishechkin 2017: 542.

#### Material examined.

None.

#### Distribution.

Sichuan (Fig. 65), western Europe to the Russian Far East including Sakhalin and Kurile Islands, Japan (Tishechkin 2017).

##### Key to species of *Oncopsis* from Sichuan Province, China based on male genitalia

**Table:** 

1	Aedeagal shaft (Figs 32, 42) strongly elongated and slender in lateral view	**2**
–	Aedeagal shaft normal, stout and typical in lateral view	**3**
2	Aedeagal shaft (Figs 32, 33) strongly tumid at middle in ventral view, and with fine protuberances on ventral margin	*** O. graciaedeagus ***
–	Aedeagal shaft (Figs 42, 43) slightly inflated at middle in ventral view, without protuberances on ventral margin	*** O. melichari ***
3	Dorsal connective process clearly bifurcated from base or sub-base	**4**
–	Dorsal connective process (Figs 13, 23) not bifurcated from base or sub-base, only apex bilobed	**13**
4	Process of dorsal connective with upper branch (Fig. 54) clearly shorter than lower one	*** O. kangdingensis ***
–	Process of dorsal connective with upper branch longer than or at least as long as lower one	**5**
5	Process of dorsal connective with upper branch (Fig. 60) clearly bent dorsad	*** O. trimaculata ***
–	Process of dorsal connective with upper branch usually bent ventrad or caudad	**6**
6	Process of dorsal connective branched from sub base	**7**
–	Process of dorsal connective branched from base	**8**
7	Inner margin between two branches of process of dorsal connective (Fig. 49) smooth, not sinuate or serrated	*** O. furca ***
–	Inner margin between two branches of process of dorsal connective (Fig. 59) serrated	*** O. nigrofasciata ***
8	Both branches of process of dorsal connective (Figs 48, 53) slender and of almost equal length	**9**
–	Upper branch of process of dorsal connective distinctly wider and shorter than lower one	**10**
9	Lower branch of process of dorsal connective (Fig. 48) bent dorsad; aedeagal shaft (Fig. 27) with lateral margins slightly sinuate in ventral view	*** O. anchorous ***
–	Lower branch of process of dorsal connective (Fig. 53) bent caudad; aedeagal shaft (Figs 34, 35) tapered to apex in ventral view	*** O. hailuogouensis ***
10	Inner margin between two branches of process of dorsal connective smooth	**11**
–	Inner margin between two branches of process of dorsal connective sinuate	**12**
11	Upper branch of process of dorsal connective (Figs 50, 51) bent ventrad and round at apex, lower branch longer than 1/2 length of upper one	*** O. fusca ***
–	Upper branch of process of dorsal connective (Fig. 55) bent caudad and subacute at apex, lower branch less than 1/2 length of upper one	*** O. kuluensis ***
12	Aedeagal shaft (Figs 40, 41) tapered in ventral view; two branches of process of dorsal connective (Fig. 56) closer to each other, upper branch sinuate and pointed caudally, and lower one slender	*** O. ludingensis ***
–	Aedeagal shaft (Figs 46, 47) with lateral parallel margins in ventral view; two branches of process of dorsal connective (Figs 61–64) away from each other, upper branch evenly bent caudally, and lower branch short	*** O. tristis ***
13	Aedeagal shaft (Figs 11, 12) tapered to apex in ventral view; process of dorsal connective (Fig. 13) with apex bifurcated and ventrally pointed	*** O. konkaensis ***
–	Aedeagal shaft (Figs 21, 22) slightly narrowed at middle in ventral view; process of dorsal connective (Fig. 23) with apex bifurcated but ventrocaudally pointed	*** O. moxiensis ***

## Supplementary Material

XML Treatment for Oncopsis

XML Treatment for Oncopsis anchorous

XML Treatment for Oncopsis furca

XML Treatment for Oncopsis fusca

XML Treatment for Oncopsis graciaedeagus

XML Treatment for Oncopsis hailuogouensis

XML Treatment for Oncopsis kangdingensis

XML Treatment for Oncopsis konkaensis

XML Treatment for Oncopsis kuluensis

XML Treatment for Oncopsis ludingensis

XML Treatment for Oncopsis melichari

XML Treatment for Oncopsis moxiensis

XML Treatment for Oncopsis nigrofasciata

XML Treatment for Oncopsis trimaculata

XML Treatment for Oncopsis tristis
